# Distinctive wound-healing characteristics in the corals *Pocillopora damicornis* and *Acropora hyacinthus* found in two different temperature regimes

**DOI:** 10.1007/s00227-016-3011-y

**Published:** 2016-10-18

**Authors:** Nikki Traylor-Knowles

**Affiliations:** 1 Hopkins Marine Station, Stanford University, 120 Oceanview Blvd, Pacific Grove, CA 93950 USA; 2 Rosenstiel School of Marine and Atmospheric Science, University of Miami, 4600 Rickenbacker Causeway, Miami, FL 33149 USA

## Abstract

Wound healing is a critical physiological function needed for survival in all marine organisms. However, it is particularly critical in organisms like corals, which cannot escape predators. In this study, I characterized the gross morphology of wound healing in *Pocillopora damicornis* and *Acropora hyacinthus* found in two pools with distinct previously documented temperature profiles in Ofu, American Samoa. I observed differences between healing rates of *A. hyacinthus* versus *P. damicornis,* but no significant difference in healing rates between *A. hyacinthus* colonies found in different environmental regimes. Both coral species exhibit very distinct healing phenotypes, where *A. hyacinthus* develops a pink pigmentation and *P. damicornis* forms an algal/sand plug. The algal/sand plug appeared in corals found in the highly variable pool more quickly than in the corals from the moderately variable pool. Lastly, *P. damicornis* appeared to never fully heal during this two-week study, indicating that it is a slower healer despite predation pressure.

## Introduction

Wound healing in corals is an important process that protects the coral from invasion by pathogens (Meszaros and Bigger 1999). When a coral is injured, it rapidly repairs the epithelial breach and regenerates lost polyps and the surrounding tissue. However, when a coral is faced with severe environmental challenges, its ability to recover from even minor injuries can be compromised (Henry and Hart 2005). Importantly, there is substantial evidence that the cumulative effects of global and local anthropogenic stressors are diminishing the ability of corals to recover from small, routine physical injuries (bites, algal abrasion, fragmentation) inflicted by predators, competitors, and pathogens (Mascarelli and Bunkley-Williams 1999; Kramarsky-Winter and Loya 2000; Hall 2001).

In general, many species of coral (including *Porites spp., Favia favus, Acropora cytherea*) heal at rates that are highest during the first stages of lesion healing and slow down due to resource limitation (Oren et al. 1997; van Woesik 1998; Downs et al. 2000). However, the actual rates of wound healing vary due to factors including species and morph, lesion size and perimeter, wound depth, and wound location (Meesters et al. 1997; Oren et al. 1997; van Woesik 1998; Downs et al. 2000; Lirman 2000; Titlyanov et al. 2005). These intrinsic factors combined with extrinsic factors such as temperature change, light changes, algal recruitment, food availability, sedimentation, and disturbance history to effect the speed at which a coral can recover and regenerate following injury (Lester and Bak 1985; Guzmán et al. 1994; Meesters et al. 1994; Oren et al. 1997; Kramarsky-Winter and Loya 2000; Downs et al. 2005).

For this article, I examined the healing rates of two different corals, *Acropora hyacinthus,* and *P. damicornis,* in the two temporally distinct back reef lagoons of Ofu Island, American Samoa, one with more variation in temperature (highly variable or HV pool) and one with less variation in temperature (moderately variable or MV pool) (Craig et al. 2001). Colonies of corals were wounded and monitored over a two-week period by digital photography. Gross anatomy of the wound was documented, and comparisons between location and species were conducted. Based on previous research, I hypothesized that: (1) *P. damicornis* and *A. hyacinthus* would have similar rates of healing (Hall 1997), (2) regeneration would occur faster in a highly disturbed habitat as opposed to a less disturbed habitat (Henry and Hart 2005), (3) faster regeneration rates for corals in higher temperatures (Denis et al. 2011), and lastly (4) *P. damicornis* would recover quickly due to the high predation pressures it experiences (Hall 1997, Henry and Hart 2005).

## Methods

### Field site and wounding assay

**Fig. 1:**
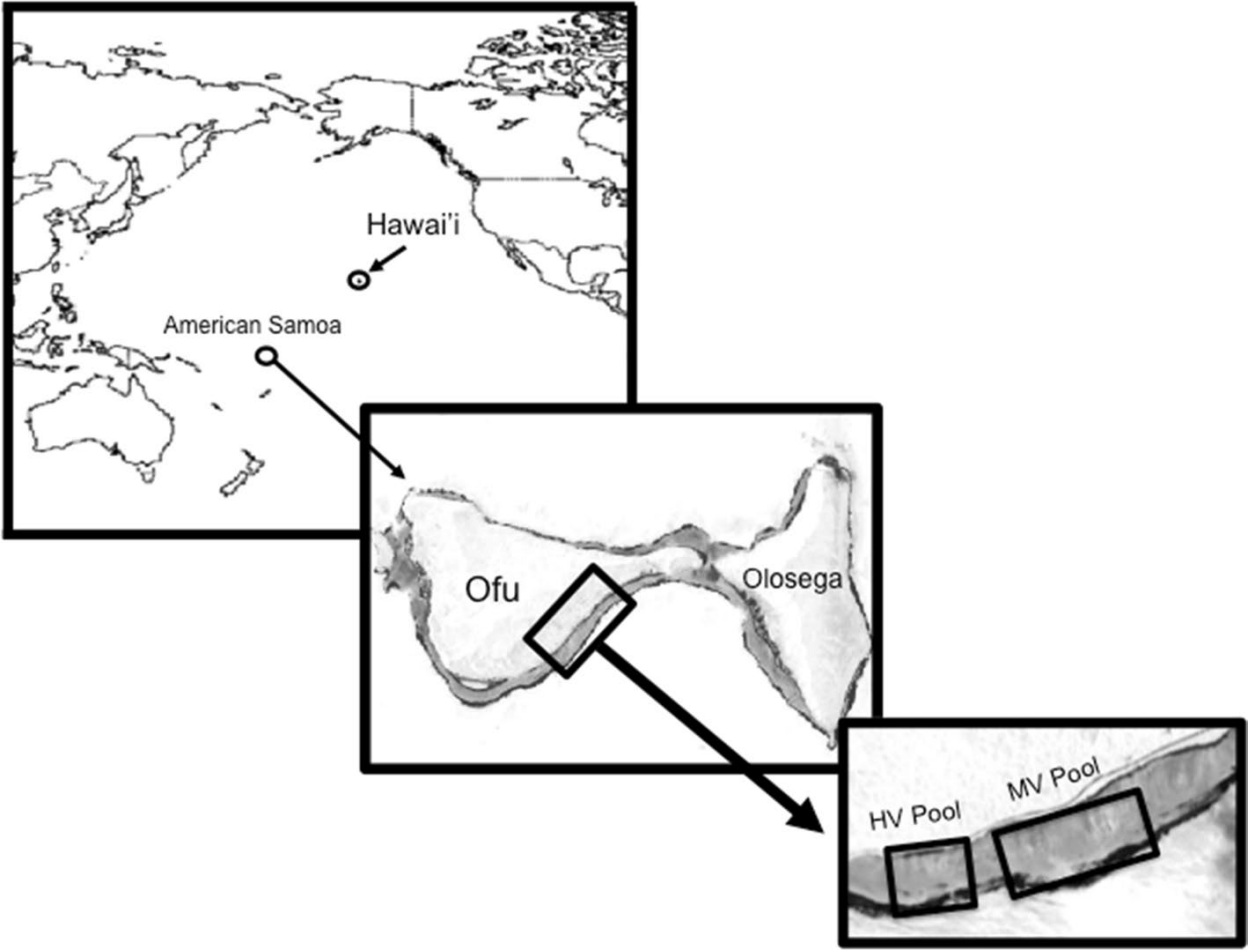
Map of American Samoa showing the location of the moderately variable pool (MV) and the highly variable pool (HV) found in Ofu Island. Ofu Island is located 14.1726°S, 169.667°W in the Pacific Ocean

**Fig. 2:**
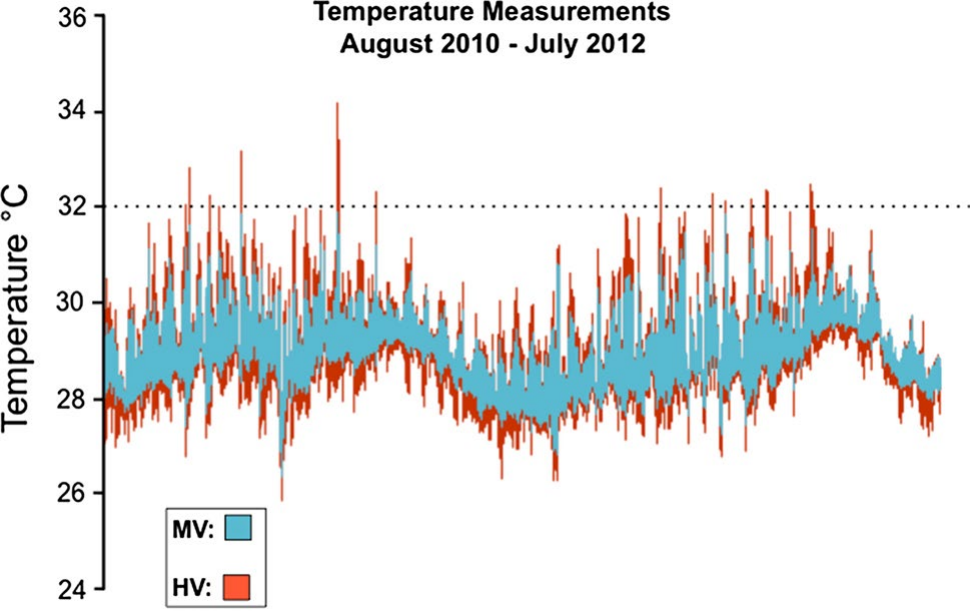
Temperature profile of the backreef lagoons of Ofu Island from August 2010 to July 2012. This shows the average of eight different data loggers within each pool that took measurements every 12 min over a two-year time. *Blue* represents the MV pool, and *red* represents the HV pool. The *dotted line* is provided at 32 °C for reference

The wounding experiments were conducted in March and April of 2012 in American Samoa in the back reef lagoons Ofu Island (Fig. 1). These pools display distinct variation in temperature, pH, and oxygen, which is primarily governed by the tidal cycle (Craig et al. 2001; Barshis et al. 2013; Ruiz-Jones and Palumbi 2014, 2015). The highly variable pool (HV pool) has high thermal variation which exceeds ≥34 °C during summer low tides and displays daily thermal fluctuations up to 6.3 °C, and the moderately variable pool (MV pool) has moderate environmental fluctuations that does not exceed the HV pool (Fig. 2) (Craig et al. 2001; Barshis et al. 2013; Ruiz-Jones and Palumbi 2014, 2015). Previously, the corals from the HV pool have been documented to have higher protein stress biomarker expression (Barshis et al. 2010), front loading of stress genes (Barshis et al. 2013), higher cellular concentration of heat-tolerant *Symbiodinium* (Oliver and Palumbi 2011), faster rates of growth (Smith et al. 2007, 2008), and more thermal tolerance (Barshis et al. 2013; Palumbi et al. 2014).

Corals were wounded by cutting off a branch from the apical branch tip of colonies of *A. hyacinthus* or *P. damicornis,* using a bone cutter. The cut size was approximately 6 mm for *A. hyacinthus* and 4 mm for *P. damicornis*. Care was taken to ensure that wound size was consistent between colonies being monitored for each species. The cut surfaces were horizontal to the overall colony due to the branch being cut away. The colonies were monitored in the field by photograph every other day, over a 2-week period, using a Pentex W-10 waterproof camera. Within the HV pool, six individual *A. hyacinthus* colonies and three individual *P. damicornis* colonies were wounded, whereas in the MV pool four colonies of *A. hyacinthus* and four colonies of *P. damicornis* were wounded and monitored.

### Data analysis

The healing rates and morphological characteristics of healing were documented by photography every other day (day 0, 2, 4, 6, 8, 10, 12, and 14) at approximately the same time, 10:00 AM SST. The healing characteristics that were monitored in *A. hyacinthus* included: (1) the appearance of tentacles and/or polyps, and (2) development of pink pigmentation with tissue cover. The day of wounding (day 0) to the day of appearance of healing characteristics was documented. While monitoring *P. damicornis,* it became clear that these metrics would not apply, so the rate at which an algal/sand plug developed was then documented. The appearance of the algal/sand plug was determined visually from photographs taken every other day. From these measurements, comparisons were done between corals from either the HV pool or the MV pool. A paired *t* test was performed in StatPlus, with a *p* value ≤0.05 considered significant.

## Results and discussion

### Overall *A. hyacinthus* and *P. damicornis* have very different methods and rates of healing

**Fig. 3:**
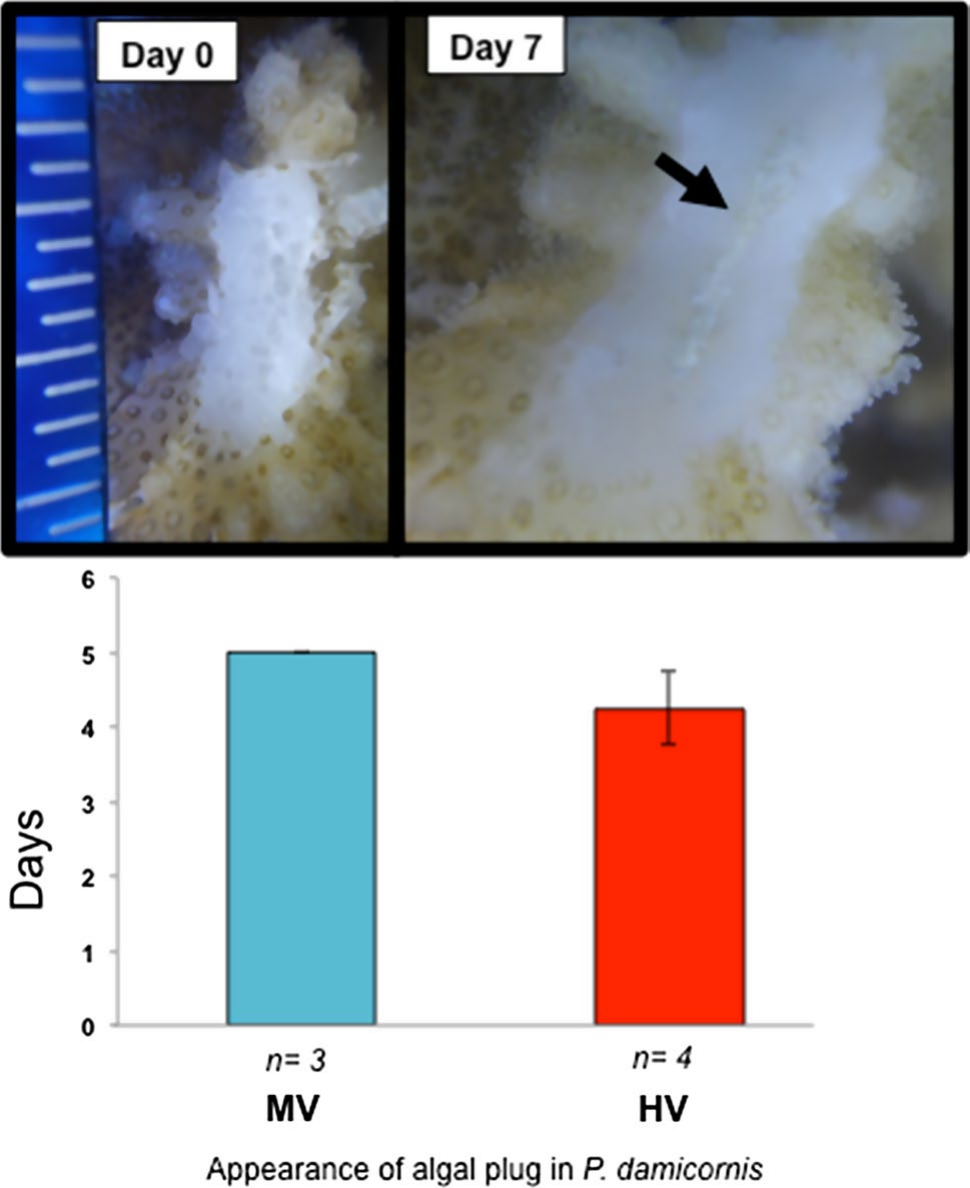
Wound-healing features found in *A. hyacinthus* in moderately variable pool (MV) and the highly variable pool (HV). *Top photographic panel* shows the healing characteristics through the experiment. The *bottom panel* shows that there was no significant difference between the colonies samples from HV and MV

Previously, *A. hyacinthus* and *P. damicornis* were found to heal at very similar rates; however, in this study, I found the contrary (Hall 1997; Henry and Hart 2005). *A. hyacinthus* healed within two weeks of wounding (Fig. 3). An average of 8 days was needed for the appearance of the polyp tentacles in *A. hyacinthus*, while pink pigmentation with tissue coverage appeared around 12.25 days (Fig. 3). Pink pigmentation was previously documented in several species of coral during wound-healing events (Palmer et al. 2009a, b), as well as in reaction to disease and tissue wasting (Palmer et al. 2009a, b). Additionally, *A. hyacinthus* showed no significant difference in the appearance of these metrics when comparing individuals from the HV pool to the individuals from the MV pool (Fig. 3). This could, however, be due to the small samples size and should be reinvestigated with larger numbers.

**Fig. 4:**
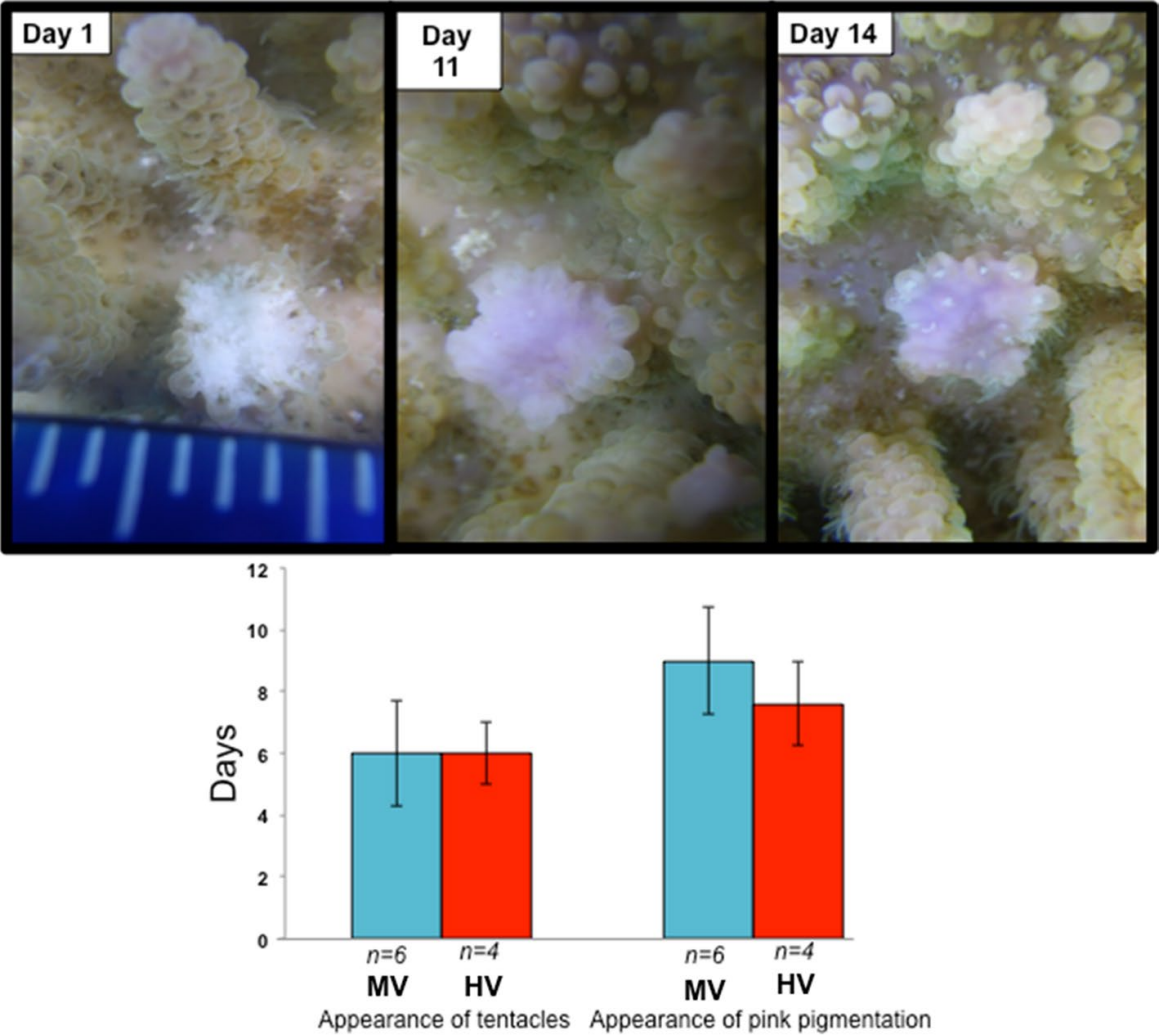
Appearance of algal/sand plug in *P. damicornis.* The *top panel* shows what the wound in *P. damicornis* looked like at Day 0 and 7 days after the wound was made. The *black arrow* is pointing to the site of the algal/sand plug. Lesion area surrounding the algal/sand plug is smooth either from tissue cells covering the area or due to erosion. The *second panel* shows that the algal plug appeared in corals from the HV pool significantly faster than ones from the MV pool. Throughout the two-week study, no changes in the algal/sand plug were observed and full healing of the wound was not observed

*P. damicornis* did not share any of the same healing characteristics with *A. hyacinthus*. Instead, these corals showed the development of an algal/sand plug in the middle of the wound site, and a clear smoothing of the skeletal area over the site of the wound (Fig. 3). This clear smoothing could be the initial covering of the skeleton by coral cells, much like what has been previously observed in gorgonian corals (Meszaros and Bigger 1999). However, because measurements were only done by photography, further work would need to be done to determine if this is truly the case. This algal/sand plug appeared on average within 4.57 days of the wounding event and remained relatively unchanged for the two-week remainder of the study (Fig. 4).

These differences in healing rate could be due to many different intrinsic and extrinsic factors including size of colony, location of colony, species of coral, and size of lesion (Bak and Steward-Vanes 1980; Chadwick and Loya 1990; Meesters and Bak 1993; Meesters et al. 1994; Van Woesik 1998; Nagelkerken et al. 1999, Lirman 2000; Henry and Hart 2005). Additionally, evolutionary differences could further confound the different healing phenotypes observed; *Pocillopora* and *Acropora* are from different clades of the coral phylogenetic tree of life (*Pocillopora* are in the Robusta clade, whereas *Acropora* are in the Complexa clade) (Kerr 2005; Stolarski et al. 2011) that are separated by approximately 415 million years of evolution (Stolarski et al. 2011). Lastly, pocilloporid corals do not have an axial polyp and do not have as deep a penetration of living tissue into the corallite as in acroporid corals (Le Tissier 1991).

### There were no differences in healing rates between *A. hyacinthus* individuals in the HV pool and MV pool

Based on previous studies, I hypothesized that colonies in the HV pool would heal more quickly than individuals in the MV pool (Denis et al. 2011). Corals that were found in areas of higher temperature had faster rates of healing, but at the cost of reproduction and growth (Henry and Hart 2005; Denis et al. 2011). I was not able to find any statistically significant differences between the rates of healing in the colonies in the MV and the HV pool. This finding, however, was limited by a small sample size of coral colonies that could be sampled and therefore warrants further investigation.

### The *P. damicornis* algal/sand plug appears sooner in HV colonies than MV colonies

Post-wounding, *P. damicornis* showed little changes in healing, except for the presence of an algal growth in the site of the wound (Fig. 4). The alga/sand plug grew from the middle of the wound area and remained this way through out the two-week monitoring period. Notably, corals that were cut in the HV pool displayed signs of the algal/sand plug sooner than corals from the MV pool, with the algal/sand plug forming in HV corals at 4.25 days and in MV corals at 5 days (Fig. 4). The formation of this algal/sand plug could be an important mechanism by which growth and recovery occurs in this coral. No pigmentation was present on the wound site. In the future, further analysis on what the source of this sand plug will need to be documented, as well as the cellular importance of this plug formation.

This plug has never been documented in the wound-healing process in *P. damicornis.* I hypothesize that this plug is a mix of tissue, algae, and sand that acts like a scab barrier during a healing event. This hypothesis would need to be tested further to understand the actual physical makeup of these plugs, and the mechanism that is controlling its development. In addition, longer monitoring times need to be done of these corals in Ofu, to observe complete healing. While previous literature has found that a related species *P. verrucosa* as well as *P. damicornis* had quick rates of regeneration due to predation pressures, this was not observed in the current study (Hall 1997; Lenihan and Edmunds 2010).

## Conclusions

In this unique system, I found evidence for wound healing to be very different in *A. hyacinthus* and *P. damicornis. A. hyacinthus* healed much more quickly, but showed no differences in healing rates between the HV corals and MV corals. *P. damicornis* did not show many characteristics of healing except for the formation of an algal/sand plug in the middle of the wound site. This plug formed more quickly in the corals found in the HV pool than corals found in the MV pool. This report presents the first evidence for a healing sand/algal plug in *P. damicornis* and could be a very important factor to healing resiliency in some corals such as *P. damicornis.*
